# Special education teachers’ mental health after reopening schools during Covid-19

**DOI:** 10.1371/journal.pone.0284870

**Published:** 2023-05-02

**Authors:** Gelan Hesham Abdou Ahmed

**Affiliations:** Curriculum and Instruction Department, Indiana University, Bloomington, IN, United States of America; Ahvaz Jundishapur University: Ahvaz Jondishapour University of Medical Sciences, ISLAMIC REPUBLIC OF IRAN

## Abstract

The purpose of this study was twofold: (a) explore special education teachers’ mental health after reopening schools during Covid-19 and (b) identify the psychological services that they needed to safeguard their mental health. In total, 10 special education teachers represented the sample of this study: three from middle schools, four from elementary schools, and three from high schools. This sample was selected using the maximal variation sampling technique. One-on-one, semi-structured interviews were carried out with the research participants. Thematic analyses of the data generated two emergent themes: stressors and psychological support. In order to safeguard special education teachers’ mental health, a personalized approach to mental health services has been recommended.

## Introduction

Due to the Covid-19 pandemic outbreak, many countries implemented tight measures, such as social distancing and city lockdowns, to decrease the imperious spread of contagion and mitigate the propagation of new infections [1–3]. With respect to the education sector, schools across the world were closed, thereby leaving more than 90% of students without any face-to-face instruction [2–5]. In conjunction with school closures, online instruction had been adopted by educational institutions worldwide to lessen the spread of Covid-19 among students and school personnel. The shift to online instruction occurred, regardless of whether teachers were prepared to employ online instruction platforms [3, 4]. In addition to the shift to online instruction, general education teachers had to integrate new methods to maintain students’ engagement during online classes and redesign educational content to facilitate their learning [1, 4–7]. For special education teachers, the situation was increasingly complicated; they were obligated to develop individualized contingency learning plans (ICLPs) to describe instructional delivery using online modality, adjust evidence-based (EB) interventions to suit online modality and train caregivers or parents to deliver them at home, inform caregivers or parents of new ways to record data so that teachers could monitor students’ progress, and set learning goals for students with disabilities that accommodate their needs during the period of online modality [8]. Indeed, these new work-related responsibilities expanded special education teachers’ workload and further aggravated their mental health.

No studies have explored the mental health of special education teachers during lockdowns. However, a large body of research has investigated the mental health of general education teachers during lockdowns [5, 7, 9–14]. For example, a qualitative study reported that teachers experienced high levels of stress and burnout because of three factors. First, lack of resources (e.g., equipment) impeded teachers from executing their roles effectively. Second, training parents to navigate online instruction platforms (e.g., Google Classroom) expanded their workload. Last, students exhibited maladaptive behaviors, including demotivation to learn, inattentiveness, and lack of autonomy and ownership [15]. Another qualitative study reported that teachers experienced heightened levels of anxiety and fear because of three facets. First, online instruction negatively affected their teaching performance, as they were ‘technologically and mentally unprepared to adapt to digital platforms’ [2, p. 217]. Second, stakeholders’ decisions about high-stakes assessments were made without any consultation with them. Last, their workload increased extensively, since they were obliged to help caregivers or parents with the pedagogical and technical aspects of their children’s home-schooling journey.

In addition to qualitative research studies, a quantitative study reported that 5% of teachers had severe burnout, 12% had moderate burnout, and 38% had mild burnout. Symptoms of burnout correlated with excessive workload, acute work problems, lack of administrative support, and use of complex information and communication technologies [16]. Another quantitative study showed that 14% of teachers had severe stress, 15% had moderate stress, and 16% had mild stress. 7% of teachers had severe anxiety, 21% had moderate anxiety, and 12% had mild anxiety. 4% of teachers had severe depression, 11% had moderate depression, and 12% had mild depression [3]. Moreover, this study reported that female teachers demonstrated higher levels of stress and anxiety (9.5% and 12.6% respectively) than male teachers (2.8% and 3.5% respectively).

After several months of closure, schools were reopened in the early fall of 2021, and face-to-face instruction was resumed [17]. Reopening schools during Covid-19 has posed an immense threat to the mental health of teachers worldwide [18]. Only two studies [e.g., 17, 19] were found that examined the mental health aspect among school personnel after reopening schools during the pandemic and focused solely on general education teachers. To begin with, Kim et al.’s study [17] reported that teachers experienced feelings of stress and anxiety because of two factors. First, their workload vastly expanded as a result of safety protocols. They were assigned to monitor students’ implementation of safety measures (e.g., disinfecting their hands and wearing face masks). Second, the uncertainty of whether schools would shut down again excessively affected their work-related responsibilities; they were incapacitated ‘to develop their lessons since they did not know when or how changes might happen’ [p. 9]. To mitigate their feelings of stress and anxiety, teachers created support networks, which empowered them to exchange solutions to work-related problems. Next, Ozamiz-Etxebarria et al.’s study [19] found that 32.2% of teachers experienced depression, 49.5% experienced anxiety, and 50.6% experienced stress. In fact, symptoms of depression, anxiety, and stress highly correlated with employment instability, heavy workload, and the possibility of being infected with Covid-19.

No studies, to my best knowledge, have investigated the mental health of special education teachers after reopening schools. Prior to Covid-19, the mental health of special education teachers was strained due to work-related responsibilities, such as modifying the individualized educational plan (IEP) of students with disabilities to include new accommodations, acquainting students with instructional and behavioral routines, assessing their socio-emotional needs constantly, monitoring their progress towards learning goals, employing EB interventions with students with disabilities, ensuring their receipt of special education services in their IEPs, and scheduling case conferences with caregivers or parents [20]. With Covid-19, another layer of strain is undoubtedly added to the mental health of special education teachers. So, the aim of the current study was to bridge the gap in the knowledge base by exploring the mental health of special education teachers after reopening schools during the pandemic, and identifying the psychological services needed to safeguard their mental health. The following research questions guided this study: (a) What are the mental health problems that special education teachers experience after reopening schools during Covid-19? (b) What are the psychological services that they need to safeguard their mental health?

## Methods

### Research design

A phenomenological research design was selected as the most suitable approach to address the two research questions, which guided this study. This is because a phenomenological approach aims to ‘describe the essence of a phenomenon by exploring it from the perspective of those, who experienced it so as to grasp the meaning participants ascribe to that phenomenon’ [21, p. 670]. In order to better explore the mental health of special education teachers after reopening schools and identify the psychological services needed to safeguard their mental health, the researcher carried out one-on-one interviews. The series of questions which guided the interviews were adapted from Kim et al.’s study [17]. Minor changes (e.g., adjusting tenses and keywords) were conscientiously made to the questions to suit the purpose of the present study.

- How can you describe your mental health after reopening schools?
- What are the factors that impacted your mental health after reopening schools?
- What kind of check-ins have you received from your school principal?
- What psychological services or interventions, if any, have you received from your school?
- What psychological services do you prefer to receive from your school?

### Procedures

Having obtained the institutional review board (IRB) approval, a formal invitation was sent to the research participants via e-mail; this invitation encompassed background information about the researcher, elucidated the aim of the study, introduced the data collection protocol, assured the anonymity of their responses, and urged them to respond to the e-mail as a means of signaling their willingness to take part in the study. Then, the research participants, who were willing to take part in the present study, were contacted via telephone to schedule a mutually agreeable date and time for the interviews. Having agreed on the interviews’ date and time, the researcher dispatched Zoom meeting links to the research participants.

At the beginning of each interview, the researcher acquainted the research participants with the aim of the study and its notable contribution to the knowledge base. Before posing the interview questions to the research participants, the researcher requested their permission to audio-record the conversation and ensured the complete anonymity of their responses. At the end of each interview, the researcher thanked the research participants for taking part in the study and for their invaluable insights.

### Ethics statement

This research project aligns with the Declaration of Helsinki and has been approved by the Human Research Protection Program (HRPP). In addition, written informed consent was obtained from all research participants.

### Inductive data analysis

Having interviewed the research participants, the researcher used Descript, a transcription software, to convert audiotape recordings into text data. To ensure that the transcription generated by Descript is free from errors, the researcher played the audiotape recordings and revised the text data. Next, the researcher conducted a preliminary exploratory analysis to comprehend the data as a whole, before breaking it into segments, and think about the organization of the data. During the preliminary exploratory analysis, the researcher read the text data, used colors to highlight similar and different ideas expressed by the research participants, and produced rudimentary ideas for data organization. Finally, the text data were uploaded into Atlas.ti, a computer-assisted qualitative data analysis software which had been previously utilized by the researcher and the coder. This software enabled the researcher and the coder to inductively code the data by ‘dividing texts into segments, labeling these segments with codes, examining them for overlap, and collapsing them into broader themes’ [22, p. 243].

### Trustworthiness

To guarantee that the data coding process was carried out conscientiously and consistently, intercoder reliability (ICR), which serves as a badge of trustworthiness, was undertaken. First, the researcher developed a coding frame, which is ‘a list of codes organized according to higher-order code categories, often accompanied by code explanations and example data segments’ [23, p. 12]. Following the recommendation of O’Conner and Joffe [23], the researcher shared the coding frame with a coder who was recruited to double code the data. At the beginning, the researcher asked the coder to double code a small amount of data (e.g., three interviews). Having established reliability through comparing the coding patterns of the researcher and the coder, and ensured the absence of any inconsistencies, the researcher requested the coder to resume double coding the remaining data (e.g., seven interviews).

### Research participants

A total of 10 special education teachers represented the sample of this study: three of which from middle schools, four of which from elementary schools, and three of which from high schools in Indianapolis. When estimating the sample size, Bekele and Ago [24] underlined that at least six research participants are required for phenomenological research studies. In addition, Hennink and Kaiser [25] postulated that the minimum sample size for achieving saturation in phenomenological research studies is six interviews. To select the sample, the researcher accessed the Monroe County Community School Corporation’s (MCCSC) staff directory page for elementary, middle, and high schools. In the elementary schools’ tab, the researcher realized that only four out of eleven schools had shifted to face-to-face instruction, while the remaining seven schools were still implementing hybrid teaching. Accordingly, the researcher selected a special education teacher from each of the four elementary schools that had fully migrated to face-to-face instruction. For the sake of the aim of the study, the researcher did not choose any special education teacher from the schools that were incorporating hybrid teaching. In the middle schools’ tab, the researcher discovered that a total of three schools had transitioned to face-to-face instruction. As a consequence, the researcher selected a special education teacher from each of the three middle schools. Finally, in the high schools’ tab, the researcher noted that only three out of six schools had adopted face-to-face instruction, whereas the remaining three schools were still utilizing hybrid teaching. Therefore, the researcher selected a special education teacher from each of the three high schools that had transferred to face-to-face instruction. Table 1 summarizes the demographic characteristics of the special education teachers.

**Table 1:** Participants’ demographics (N = 10).

Characteristic	Ratios (n)
**Gender**	
Female	0.7 (7)
Male	0.3 (3)
**Highest Educational Degree**	
Bachelor’s degree	0.8 (8)
Master’s degree	0.2 (2)
Ph.D.	0 (0)
**Years of Teaching Experience**	
0–5 years	0.4 (4)
5–10 years	0.3 (3)
10–15 years	0.2 (2)
Above 15 years	0.1 (1)
**Grade Levels**	
Elementary	0.4 (4)
Middle	0.3 (3)
High	0.3 (3)
**Age**	
20–30 years	0.3 (3)
30–40 years	0.3 (3)
40–50 years	0.1 (1)
Above 50 years	0.3 (3)

The aforementioned sample was selected using the maximal variation sampling technique; maximal variation sampling is ‘a purposeful sampling technique, in which the researcher samples cases, or individuals that differ on some characteristic or trait’ [22, p. 208]. The researcher utilized the maximal variation sampling technique to capture the different perspectives of special education teachers, who are currently working in elementary, middle, and high schools in Indianapolis, USA, about their mental health after reopening schools amidst Covid-19, and the psychological services needed to safeguard their mental health.

## Findings

Thematic analyses of the data generated two emergent themes: stressors and psychological support. Table 2 associates the research questions that guided this study with the major themes and subthemes, emerging from the participants’ responses.

**Table 2:** Research questions, major themes, and subthemes.

Research Questions	Major Themes	Subthemes
1- What are the mental health problems that special education teachers experience after reopening schools?	Stressors	a) Students’ behaviors b) Safety measures c) IEP responsibilities d) Uncertainty e) Contact Tracing
2- What are the psychological services that they need to safeguard their mental health?	Psychological support	a) Social support b) Schools’ support c) Preferred support

### Stressors

After reopening schools, all special education teachers exhibited feelings of stress because of a number of factors. These factors are outlined in the following subthemes: students’ behaviors, safety measures, IEP responsibilities, uncertainty, and contact tracing. These subthemes have been organized from the most frequent to the least frequent.

#### Students’ behaviors

One of the most frequently reported factors, causing stress to all special education teachers, was students’ behaviors. After reopening schools, special education teachers reported an immense decline in students’ social skills. Across different grade levels, students struggled to communicate and interact with their colleagues, and engage in active listening with others. For example, a special education teacher stated “the biggest thing that I have seen from kids is a low window of tolerance within the classroom. They talk out of turn. They are unable to interact with one another.” Further, all special education teachers noticed that students lacked school readiness skills, like maintaining focus on a task. Accordingly, more than half of instructional time was spent on refocusing students on the task at hand. For instance, a special education teacher said “they cannot pay attention to me for longer than 10 minutes; they were used to being online and having these chunks of free time.”

#### Safety measures

The second most frequently reported factor, triggering stress to special education teachers, was safety measures. After reopening schools, all special education teachers implemented a series of safety measures, including sanitizing their hands, maintaining social distance, and wearing face masks, to diminish the spread of Covid-19. For example, a special education teacher stated “there are a lot of those protocols that add stress. You know, outside of just what you are already worrying about. You have to keep distant and to use sanitization.” Besides, special education teachers were responsible for ensuring students’ implementation of such measures to mitigate the propagation of new infections. For instance, a special education teacher said “you have to make sure [the students] have their masks on and are six feet apart. So, that is another layer of stress.” Further, some special education teachers discussed how safety measures impinged their instructional strategies. Because students had to be six feet apart, special education teachers refrained from employing instructional practices that promoted collaborative work. For example, a special education teacher stated “hence instruction changed. I do large group instruction all the time. Safety measures altered the way you had to teach and narrowed down your educational strategies.”

#### IEP responsibilities

IEP responsibilities were one of the least referenced factors, bringing about stress to special education teachers. Only one special education teacher tackled IEP responsibilities in his interview responses. This teacher exerted major efforts to ensure that students with emotional and behavioral disorders (EBD) received the exact number of counseling sessions listed in their IEPs after schools were reopened. During school closures, students with EBD received a few counseling sessions due to the pandemic. For example, a special education teacher said “I made sure to achieve the services in students’ IEP. I contacted service providers during school closures and agreed on a less number. But now, I am making sure that the number of sessions in their IEPs is met.”

#### Uncertainty

Uncertainty was also one of the least referenced factors, causing stress to special education teachers. Only one special education teacher experienced feelings of uncertainty after schools were reopened. With the surge in Covid-19 cases and the appearance of the Omicron variant, this special education teacher was unsure of whether the government would force nationwide school closures, or would keep them open. These feelings of uncertainty impacted his duties as a teacher, especially when it came to developing lesson plans and assignments. For instance, a special education teacher said “the uncertainty was stressful for planning. It was hard to plan more than one week in advance because we did not know if next week our numbers would go up and it would shut the school down again.”

#### Contact tracing

Contact tracing was too one of the least referenced factors, bringing about stress to special education teachers. Only one special education teacher discussed the contact tracing process in his interview responses. After reopening schools, this teacher was responsible for developing a seating chart for his students. This chart enabled him to make quarantine decisions for students, who were in contact with a Covid-19 infected classmate. Also, he ensured that the return dates for those, who were quarantined, were consistent. For example, a special education teacher said “I would have to turn in a seating chart. Anytime someone was to become positive in my room, I would have to go around and see who was around those students for upwards of 15 minutes.”

### Psychological support

This theme provides a detailed account of the psychological support that special education teachers received from their colleagues and schools. Also, it presents a comprehensive description of their preferred psychological services. This theme encompasses the following subthemes: social support, schools’ support, and preferred support.

#### Social support

After reopening schools, all special education teachers reported not receiving mental health check-ins from their principals (e.g., asking them how they are feeling today and if they are sensing any symptoms of depression, stress, and/or anxiety). For example, a special education teacher said “there were no mental health check-ins from our school administration. They have not really done much of anything to us.” However, all special education teachers asserted receiving newsletters or emails from their school administration about effective exercises and practices that could improve their mental health. For instance, a special education teacher stated “they send us many things that we could do. We get an e-mail saying go to this site and practice breathing exercises.” In addition, all special education teachers emphasized receiving mental health check-ins from their colleagues on a daily basis. During break time, special education teachers approached their colleagues in their classrooms to make sure that they were feeling well, to provide aid and support in any work-related duties, and to exchange solutions to classroom problems. For example, a special education teacher said “we care about each other. You see someone who has that look on their face and you ask them how they are doing and you listen for a while.”

#### Schools’ support

After reopening schools, all elementary and middle school special education teachers were provided with psychological services. These services are an essential part of their health insurance plan, offered by their schools. For instance, a special education teacher said “we have access to six free counseling sessions per year. They are part of our basic medical package.” Although they had access to psychological services, several elementary and middle school special education teachers reported not making use of them because of a myriad of reasons, including the low-quality care of several psychological services’ centers, the limited working hours of counselors, and their distant locations. Unlike elementary and middle school special education teachers, all high school special education teachers were not allocated any psychological services from their schools. For example, a special education teacher stated “it would have been nice to be provided with any psychological services after reopening schools; however, I was not offered anything.” Further, many high school special education teachers associated the lack of mental health support with high teacher turnover rates. As their mental health was not a priority to school principals, special education teachers were continuously leaving the profession. For instance, a special education teacher stated “statistics tell us that teachers are leaving special education, so [school principals] should offer something to us. It would be nice if there were mental health services that were more readily available.”

#### Preferred support

All special education teachers proposed several psychological services that they longed to receive from their schools. These services would help decrease the stress associated with reopening schools. One of the proposed psychological services was yoga. Several special education teachers suggested having weekly yoga classes on school campus; these classes would help them meditate and achieve mindfulness. For instance, a special education teacher said “if somebody came in once a week and led yoga classes, that would be excellent for our mental health.” Another psychological service that was proposed by some special education teachers was on-campus counselors. Having on-campus counselors would provide teachers with instantaneous opportunities to dialogue with a professional about their mental health concerns. For example, a special education teacher reported “if on-campus counseling could be offered to us, this would be phenomenal. Counselors would be checking in with us to make sure we are able to perform.” The last proposed psychological service was group therapy. Several special education teachers emphasized that group therapy would highly enable them to develop a support network, exchange ideas to improve a challenging situation, and share positive coping mechanisms. For instance, a special education teacher stated “group therapy enables you to talk to other people who may be going through what you are going through or share ideas and advice with individuals.”

## Discussion

The current study was conducted to explore special education teachers’ mental health after reopening schools. The sample comprised a total of 10 special education teachers who are currently tutoring different grade levels in public schools in Indianapolis, U.S.A. In effect, thematic analyses of the data generated two major themes: stressors and psychological support. To begin, all special education teachers experienced feelings of stress after reopening schools because of many factors, such as students’ behaviors, safety measures, IEP responsibilities, uncertainty, and contact tracing. These feelings of stress were echoed in a research study which probed general education teachers’ mental health after reopening schools and reported that 50.6% of the teachers suffered from stress [19].

One major source of stress to special education teachers was students’ behaviors. In effect, special education teachers observed a severe decline in students’ social and school readiness skills. This observation was consistent with a recent study which investigated general education teachers’ mental health during lockdowns and indicated that teachers experienced feelings of stress because of students’ behaviors. Students were reported to lack autonomy and ownership, to be demotivated to learn, and to have a minimal attention span [15]. Even though this study was carried out during lockdowns and included a different population of teachers, it was evident that students’ behaviors were not a characteristic of periods of lockdown.

A second major source of stress to special education teachers was safety measures. Indeed, special education teachers were required to implement safety measures on school campus, such as wearing face masks, maintaining social distance, and sanitizing their hands. In addition, they were responsible for monitoring students’ exercise of safety measures. This source of stress was echoed in a recent study which scrutinized the prevalence of depression, stress, and anxiety among general education teachers after reopening schools and reported that they suffered from symptoms of stress because of safety measures [17]. Teachers were required to implement safety measures inside their classrooms (e.g., sanitizing students’ desks between classes) to diminish the spread of Covid-19.

One of the infrequent, yet conducive sources of stress was uncertainty. With the increasing infectivity rates and the appearance of the Omicron variant, a special education teacher was unsure of whether schools would shut down again or remain open. These feelings of uncertainty resonated with a recent study which assessed the symptoms of depression, stress, and anxiety among general education teachers after reopening schools and underlined that they experienced stress because of uncertainty. They were unable to execute their job-related duties (e.g., developing lesson plans and assignments), as they ‘did not know when or how changes may happen’ [17, p. 9]. They were also frustrated by ‘the government’s last-minute decisions and the schools’ ever-changing expectations about what [they] should be doing” [p. 8].

Another infrequent, yet causative source of stress was IEP responsibilities. After reopening schools, a special education teacher suffered from stress while revisiting the IEPs of students with disabilities and guaranteeing their receipt of special education services. A possible cause for these feelings of stress could be the lack of administrative support. A recent study which scrutinized the prevalence of depression, stress, and anxiety among general education teachers during lockdowns reported that they suffered from stress due to the absence of administrative support in work-related aspects, such as compiling records, and preparing documents and reports [3].

The last infrequent, yet contributing source of stress was contact tracing procedures. When schools were reopened, a special education teacher was in charge of developing a seating chart for his class; this seating chart informed his quarantine-related decisions and enabled him to keep track of students’ return dates. A plausible reason for the infrequency of this subtheme could be that the Indiana Department of Health [26], as of February 23^rd^, ‘no longer recommends contact tracing in schools’ [p. 12]. Yet, schools, in partnership with Local Health Departments (LHDs), may resume contact tracing and quarantine protocols. Since the interviews were launched on April 1^st^, most of the schools in Indianapolis may have ceased contact tracing and quarantine measures.

The second major theme was psychological support. It consists of the following subthemes: social support, schools’ support, and preferred support. To start with, all special education teachers emphasized that they did not receive any mental health check-ins from their school principals. Yet, they received mental health check-ins from their colleagues. This form of mental health check-ins was referenced in a recent study in which general education teachers postulated that social support ‘was a protective factor of their mental health and well-being’ [17, p. 14]. In fact, developing social networks enabled them to discuss work-related issues, share coping techniques, seek support, and exchange solutions to classroom problems.

The next subtheme was schools’ support. In fact, all elementary and middle school special education teachers had access to six free counseling sessions as a vital part of their schools’ health insurance plan. According to the UNESCO [27], these sessions aim at enabling teachers to actively support their mental health and their students’. Compared to elementary and middle school special education teachers, all high school special education teachers were not allocated any psychological services from their schools. Failure to provide teachers with psychological services can negatively affect the quality of their teaching performance, thus jeopardizing students’ academic achievement [28] and increasing teacher absenteeism [15].

The last subtheme was preferred support. Special education teachers proposed a number of psychological services, including yoga classes, group therapy, and on-campus counseling sessions, that they yearned to receive from their schools. These services would help them diminish the stress associated with reopening schools. Demonstrating genuine concern for teachers’ mental health by listening and responding to their psychological needs augments their effectiveness, increases their productivity, promotes their psychological wellness, and creates a positive work environment and atmosphere [29].

### Study limitations and future research

The present study has some limitations. First, given the fact that there are scarce studies on teachers’ mental health during lockdowns and after reopening schools, there is a high risk of citing literature with moderate-to-low methodological quality. Second, all the special education teachers, recruited in this study, belonged to only one racial group (e.g., White). Hence, future studies need to explore the mental health of special education teachers, who are racially diverse (e.g., Black and Hispanic), after reopening schools and identify their preferred psychological services. Last, all the special education teachers, at the time of the interviews, were working in public schools. Therefore, future studies need to investigate the mental health of special education teachers, who are working in private schools, after reopening schools and pinpoint their preferred psychological services.

### Implications for policy

Stress is a primary predictor of special education teachers’ early retirement or resignation [30]. Due to stress, the number of special education teachers, who planned to leave the profession or retire early, in Indianapolis rose from 28% to 55%, thereby exacerbating the problem of teacher shortages [31]. In effect, supporting special education teachers’ mental health is key in combating the problem of early retirements and resignations [18, 27]. Instead of the standardized approach to mental health care whereby teachers receive pre-existing support systems in the form of counseling sessions that may or may not meet their needs, mental health services need to be personalized [32]. Personalization, a novel approach to service delivery in the mental health arena, is deemed as ‘the way in which services are tailored to the needs and preferences of individuals’ [32, p. 5].

A personalized approach to mental health, as accentuated by Mind [32], includes two main phases: person-centered needs assessment and self-directed support. Firstly, in the person-centered needs assessment, the mental health of individuals is screened so that they grasp and better address their needs. Secondly, individuals, in the self-directed support, create their own support plans and choose the services that align with their preferences and needs. Indeed, personalizing mental health services has been documented to improve individuals’ path to recovery and to manage their mental health problems [33].
